# Positive Events and Physical Activity in Daily Life for Middle-Aged and Older Adults

**DOI:** 10.1093/geroni/igab046.163

**Published:** 2021-12-17

**Authors:** Sun Ah Lee, Lizbeth Benson, Erica O'Brien, David Conroy, David Almeida

**Affiliations:** 1 The Pennsylvania State University, University Park, Pennsylvania, United States; 2 The University of Oklahoma Health Sciences Center, United States; 4 Pennsylvania State University, University Park, Pennsylvania, United States

## Abstract

Previous studies reveal that positive affective well-being is positively associated with physical activity. The present study extends this work by examining the relationship between positive events and physical activity in daily life. Participants (N=1,016, ages 43-90, 56% women) from the third wave of the National Study of Daily Experiences reported their experiences of positive events and physical activity in eight daily diary interviews. Results from multilevel model analyses showed that on days when participants experienced more positive events than usual, they were more likely to engage in physical activity and reported engaging in greater physical activity than usual. Further, participants who experienced more positive events on average across the study period also reported engaging in greater daily physical activity. These results were invariant across age. Our findings highlight the importance of naturally-occurring positive experiences in daily life across middle- and later adulthood.

